# Seroprevalence of anti-SARS-CoV-2 antibodies in household domestic ferrets (*Mustela putorius furo*) in Spain, 2019–2023

**DOI:** 10.1007/s11259-023-10190-2

**Published:** 2023-08-07

**Authors:** Jacobo Giner, María Eugenia Lebrero, Michele Trotta, Pablo Rueda, Laura Vilalta, Maite Verde, Ramón Hurtado-Guerrero, Julián Pardo, Delia Lacasta, Llipsy Santiago, Maykel Arias, Natacha Peña-Fresneda, Andrés Montesinos, María D. Pérez, Antonio Fernández, Sergio Villanueva-Saz

**Affiliations:** 1 Clinical Immunology Laboratory, Veterinary Faculty, University of Zaragoza, 50013 Zaragoza, Spain; 2 Department of Animal Pathology, Veterinary Faculty, University of Zaragoza, Zaragoza, Spain; 3 Instituto Agroalimentario de Aragón-IA2 (Universidad de Zaragoza-CITA), Zaragoza, Spain; 4 Hospital Veterinari Canis, Girona, Spain; 5 Institute for Biocomputation and Physics of Complex Systems (BIFI), University of Zaragoza, Edificio I+D, Campus Rio Ebro, Zaragoza, Spain; 6 Aragon I+D Foundation (ARAID), Zaragoza, Spain; 7 Aragon Health Research Institute (IIS Aragón), Zaragoza, Spain; 8 Department of Microbiology, Pediatrics, Radiology and Public Health, University of Zaragoza, Zaragoza, Spain; 9 CIBER de Enfermedades Infecciosas, Instituto de Salud Carlos III, Madrid, Spain; 10 Department of Animal Medicine and Surgery, Veterinary Faculty, Univerisdad Complutense Madrid, Madrid, Spain; 11 Hospital Veterinario de Animales Exoticos Los Suaces, Madrid, Spain; 12 Department of Animal Production and Sciences of the Food, Veterinary Faculty, University of Zaragoza, Zaragoza, Spain

**Keywords:** ELISA, Ferret, SARS-CoV-2, Serology, Spain, VNT

## Abstract

SARS-CoV-2 is the causal agent of Coronavirus Disease 2019 (COVID-19) in humans that emerged in late 2019. This virus is able to infect humans and different animal species. Among pets, cats and ferrets are more susceptible to be infected by the SARS-CoV-2. Epidemiological studies are an important tool to provide information under natural conditions of exposure to SARS-CoV-2 virus. In comparison to cats, limited epidemiological studies have been performed in domestic ferrets (*Mustela putorius furo)* reporting the presence of antibodies in this species. This study analysed the presence of anti-SARS-CoV-2 antibodies in 432 cliend-owned ferrets from different geographical areas of Spain during the different waves of COVID-19 outbreaks from December 2019 to May 2023 (42 months). For this purpose, anti-SARS-CoV-2 antibodies were detected by an enzyme-linked immunosorbent method (ELISA) using the receptor binding domain (RBD) of Spike antigen and confirmed by serum virus neutralization assay. Eighteen of the 432 ferrets included were seroreactive by the in-house ELISA (4.17%, 95% Confidence Interval (CI): 2.65–6.49). In this sense, the wave of COVID-19 with the higher number of seropositive ferrets occurred during the seventh wave when the different Omicron subvariants were the dominant virus variants. Our results suggest that the risk of SARS-CoV-2 transmission in domestic ferrets in natural conditions is low. Further research is need to evaluate the potential risk of transmission of SARS-CoV-2 from human to pets.

**Supplementary information:**

The online version contains supplementary material available at 10.1007/s11259-023-10190-2.

## Introduction

Severe Acute Respiratory Syndrome Coronavirus 2 (SARS-CoV-2) is the etiologic virus responsible for Coronavirus Disease 2019 (COVID-19), which was detected for the first time in China in December 2019. This virus has a zoonotic origin and different domestic and wildlife animal species are susceptible to the infection. Virus susceptibility is associated with the ability to bind the animal/human host angiontensin-converting enzyme 2 by the Receptor-Binding Domain (RBD) of SARS-CoV-2 Spike protein (Gryseels et al. 2021).

Human-to-animal transmission is the main route in the case of domestic animal although transmission animal-to-animal could occur by direct contact. The means of this transmission are usually through respiratory droplets and secretions, nevertheless the presence of aerosols may be implied. Among companion animals, susceptibility to the infection has been demonstrated by experimental and natural conditions in cats, dogs and ferrets. In the particular case of ferrets, high virus replication in the upper respiratory tract and infectivity in conjunction with the presence of clinical signs have been detected in experimental infection suggesting a potential reservoir of this host (Schlottau et al. 2020; Shi et al. 2020).

There are some gaps in the knowledge in SARS-CoV-2 epidemiology in ferrets in natural conditions in comparison to dogs and cats. On the other hand, the high susceptibility to the virus in minks, which belong to the same family as ferrets, Mustelidae family, is well documented as well as the transmission of mink-to-human (Oude Munnink et al. 2021). For this reason, the lack of extensive knowledge related to domestic ferrets and SARS-CoV-2 becomes relevant in order to assess the possibility of this species to be a possible reservoir of the virus taken into account that the number of ferrets as pets is increasing (Dancer et al. 2022).

Epidemiological studies are relevant to investigate the prevalence of SARS-CoV-2 infection in animal hosts. Detection of anti-SARS-CoV-2 antibodies in a serum sample is indicative of exposure of the host immune system to the SARS-CoV-2 virus. For this purpose, different techniques are available to detect the presence of anti-SARS-CoV-2 antibodies. Among serological techniques, enzyme-linked immunosorbent assay and virus neutralization test (VNT) are the most common technique performed in sero-epidemiological studies. In the case of ELISA technique, the plate could be coated with different types of antigens including the whole spike protein (S), the viral nucleocapsid (N) or membrane (M) proteins and RBD of spike, being the most common antigens used nucleocapsid and RBD of Spike (Villanueva-Saz et al. 2022). Finally, VNT is considered as the gold standard technique to evaluate the interaction between anti-SARS-CoV-2 neutralizing antibodies and the SARS-CoV-2 in vitro conditions. An important difference between ELISA technique and VNT is detected because in the case of the ELISA adapting and validating the technique to each species studied is necessary, whereas in the VNT the neutralizing activity of antibodies is species-independent (Saker et al. 2022).

Different epidemiological studies in dogs and cats in different conditions have been published including case of series, cross-sectional surveys and large-scale studies in Europe and other non-European countries. Epidemiological information related to ferrets is limited to three epidemiological studies with a few numbers of samples, one from Poland and two from Spain (Giner et al. 2021; Gortázar et al. 2021), as well as the description of two clinical cases, one detected in the EEUU (APHIS 2021) and another case detected in Slovenia (Sta 2020).

Given the limited number of ferret SARS-CoV-2 infection epidemiological studies, the purposes of the present study are: (1) to investigate the seroprevalence of SARS-CoV-2 infection in different regions of Spain using RBD-based ELISA and confirmed by serum virus neutralization assay; and (2) to analyze the presence of variation of the seropositivity from the different waves of COVID-19 outbreaks.

## Materials and methods

### Animals

Four hundred and thirty-two client-owned ferrets serum samples were obtained. Sampling comprised ferrets under the care of private owners including seventeen Provinces in Spain during the different waves of COVID-19 outbreaks from December 2019 to May 2023. The inclusion of the ferrets was random and based on the owner’s decision as to whether stored serum samples of their animals may be used for SARS-CoV-2 serology or residual samples obtained for other diagnostic purposes. In all cases, serum samples were obtained with the consent of the owner to research purposes. Before sampling, information was obtained about each animal regarding age, gender, neutering (intact, surgical castration/spaying, hormone implant), cohabitation with other animals, health status prior to blood collection (sick versus healthy), lifestyle (indoor, outdoor, or mixed), location and date of collection. A volume of five hundred microliters of blood were collected aseptically by cranial cava venipuncture from each ferret. The blood was left in tubes and after clotting, a centrifugation at moderate speed (450 g) was performed to obtain the serum. Finally, the serum was taken, placed in a storage tube and stored at -20ºC until processing. In the case of residual serum samples from patients seen for medical reasons or routine healthcare check-ups, information includes location and date of collection and these samples were store at -20ºC until processing.

### Detection of SARS-CoV-2 antibodies IgG (anti-RBD) by in-house ELISA

Antibodies to SARS-CoV-2 were determined by an indirect ELISA for the detection of IgG specific for RBD (Ancestral SARS-CoV-2 strain, Wuhan strain), described previously (Giner et al. 2021; Villanueva-Saz et al. 2022). Ninety-six–well plates were coated overnight, at 4 °C with 100 ng RBD protein in phosphate-buffered saline (PBS). Subsequently, the coating solution was removed and the plate was washed three times with 200 µL per well of PBS + TWEEN 0.05% (PBST). 300 µL of PBST containing 3% dry skimmed milk was added to each well as blocking solution. The plate was incubated with blocking solution for 1 h at 37 °C in a moist chamber. 100 µL of ferret sera, diluted 1:100 in PBS containing 0.05% Tween 20 and 1% dry skimmed milk (PBST-M), was added to each well. The plates were incubated for 1 h at 37 °C in a moist chamber. After washing the plates for 30 s 6 times with PBST followed by 1 wash with PBS for 1 min, 100 µL/well of Protein A/G conjugated to horseradish peroxidase (Thermo Fisher Scientific, Waltham, MA, USA) diluted 1:100,000 in PBST-M was added per well. The plates were incubated for 1 h at 37 °C in a moist chamber and were washed again with PBST and PBS as described above. The substrate solution (ortho-phenylene-diamine) and stable peroxide substrate buffer (Thermo Fisher Scientific, Waltham, MA, USA) were added at 100 µL per well and developed for 20 ± 5 min at room temperature in the dark. The reaction was stopped by adding 100 µL of 2.5 M H_2_SO_4_ to each well. Absorbance values were read at 492 nm. in an automatic micro ELISA reader (ELISA Reader Labsystems Multiskan, Midland, ON, Canada). As a positive control, each plate included serum from a seropositive ferret detected previously (Giner et al. 2021), and serum from a healthy, non-infected ferret obtained prior to pandemic COVID-19 pandemic situation as negative control. The same positive and negative sera were used for all assays. All samples were run in duplicate The cut-off was set to 0.250 Optical Density units (OD units) (mean + 3 standard deviations of values from 61 ferrets obtained prior to the COVID-19 situation as negative controls) and the results above this value were considered positive. Control sera were obtained from the collection of sera of the Laboratory of Clinical Immunology at the Faculty of Veterinary Medicine, University of Zaragoza, Spain.

### Micro-neutralization assay of SARS-CoV-2

This virus neutralization test (VNT) was performed as described previously (Villanueva-Saz et al. 2023a). The viral strain used for this technique was the B1.1 strain (Alpha variant, B.1.1.7). The neutralization ID50 was calculated as the highest dilution that protected more than 50% of the wells from cytopathic effect. This test was performed in serum samples that tested positive in the in-house ELISA for SARS-CoV-2 antibody detection as complementary technique.

### Statistical analysis

Data collection for the entire population were analyzed using descriptive analysis. Univariate analysis of categorical data was performed to determine possible associations between SARS-CoV-2 seropositivity and the following variables: age, gender, neutering, cohabitation with other animals, health status during the blood collection, lifestyle, and date of collection were analysed. Missing observations were excluded from the analysis. The significance of this difference was assessed using the Chi-Square or Fisher´s exact test. A value of p ≤ 0.05 was considered significant. The statistics were performed using SPSS version 22.0 statistical package (IBM Corporation, Armonk, NY, USA).

## Results

### Epidemiological characterization of the ferrets

In total, 432 client-owned ferrets were sampled. Of the 432 samples, 114 were residual samples obtained for other diagnostic purpose, and 318 remaining samples were collected from ferrets with epidemiological information. All the tested ferrets (*n* = 318; 147 females and 171 males) had a mixture of coat colors. Of the 318 ferrets included, they were classified as young (≤ 1 year), adult (from > 1 year to < 5 years), or senior (≥ 5 years) (Table 1).

**Table 1:** Evidence of exposure with SARS-CoV-2 in ferrets using ELISA

Variable	ELISA	
	Positive	Negative
Gender		
Female Male Total	8 6 14	139 165 304
Age		
Young Adult Senior Total	1 6 7 14	54 126 122 302
Lifestyle		
Indoor Outdoor Mixed Total	7 0 6 13	211 11 73 295
Neutering		
Intact Surgical castration/spaying Hormone Implant Total	2 1 11 14	89 68 142 299
Cohabitation with animals		
Yes No Total	2 11 13	87 177 264
Health status		
Healthy Sick Total	6 8 14	137 163 300

No significant association (*p* > 0.05) was detected between SARS-CoV-2 seropositivity by ELISA and age (young, adult, senior), gender (male/female), neutering (intact, surgical castration/spaying, hormone implant), cohabitation with animals, health status prior to blood collection (sick versus healthy), or lifestyle (indoor, outdoor, or mixed). A significant association was detected between waves of COVID-19 outbreak and SARS-CoV-2 seropositivity by ELISA (*p* = 0.002) (Supplementary Table 1).

Related to the location of the samples included in this study, the number of samples was variable depending the Province, being the Valencia (*n* = 143), followed by Madrid (*n* = 140) with the higher number of samples collected. By contrast, Coruña (*n* = 1), Burgos (*n* = 2), Barcelona (*n* = 2), Castellon (*n* = 2), Huelva (*n* = 2) were the Provinces with the lowest number of samples included in the study (Table 2).

**Table 2:** Number of ferrets sampled included in this study from each Province detected by ELISA

Spanish Province	Number of samples collected	Number of positive ferrets	Seroprevalence (95% confidence interval)
Alava	18	1	5.56 (95% CI 0.99–25.76)
Alicante	6	0	0.00 (95% CI 0.00 -39.03)
Barcelona	2	0	0.00 (95% CI 0.00-65.76)
Vizcaya	20	3	15.00 (95% CI 5.24–36.04)
Burgos	2	0	0.00 (95% CI 0.00-65.76)
Castellon	2	0	0.00 (95% CI 0.00-65.76)
Coruña	1	0	0.00 (95% CI 0.00-79.35)
Gerona	18	0	0.00 (95% CI 0.00-17.59)
Las Palmas	19	1	5.26 (95% CI 0.94–24.64)
Huelva	2	0	0.00 (95% CI 0.00-65.76)
Madrid	100	3	3.00 (95% CI 1.03–8.45)
Murcia	4	0	0.00 (95% CI 0.00-48.99)
Navarra	5	3	60.00 (95% CI 23.07–88.24)
Pontevedra	8	0	0.00 (95% CI 0.00-32.44)
Sevilla	28	2	7.14 (95% CI 1.98–22.65)
Valencia	143	3	2.10 (95% CI 0.72–5.99)
Zaragoza	54	2	3.70 (95% CI 1.02–12.54)
Total of samples	432	18	4.17 (95% CI 2.65–6.49)

### Serological prevalence of SARS-CoV-2 infection

Eighteen of the 432 ferrets included were seroreactive by the in-house ELISA (4.17%, 95% CI: 2.65–6.49) including 6 males, 8 females and 4 non-determined. The OD units ranging from 0.261 to 0.519 (cutoff ≥ 0.250). None of the seropositive to SARS-CoV-2 ferrets were positive to VNT (Supplementary Table 2).

Considering the different waves of COVID-19 outbreaks, seropositive ferrets were detected in all waves except in the fourth wave (Table 3). It is important to remark that after the seventh wave, a seropositive ferret was also detected. The wave of COVID-19 with the higher number of seropositive ferrets occurred during the seventh wave (17.65%, 95% CI 9.57–30.25), followed by the sixth wave (5.88%, 95% CI 1.05–26.98), the second wave (5.00%, 95% CI 0.89–23.61), the fifth wave (4.00%, 95% CI 1.10-13.46). The remaining waves of COVID-19, the seroprevalence was less than 3.00% (Table 3). Significant differences were detected between the first wave and the seventh wave (*p* = 0.002), third wave and seventh wave (*p* = 0.008) and seventh wave and the samples collected after the seventh wave (*p* = 0.017) (Supplementary Table 3).

**Table 3:** Number of samples collected and collection time

Number waves of COVID-19 outbreak	Collection time	Prevalent SARS-CoV-2 variant in Spain	Number of samples collected	Number of positive samples	Seroprevalence (95% confidence interval)
First wave	From December 2019 to September 2020	Ancestral SARS-CoV-2 strain	103	2	1.94 (95% CI 0.53–6.81)
Second wave	From October to December 2020	Ancestral SARS-CoV-2 strain	20	1	5.00 (95% CI 0.89–23.61)
Third wave	From January to April 2021	Ancestral SARS-CoV-2 strain - Alpha (B.1.1.7)	82	2	2.44 (95% CI 0.67–8.46)
Fourth wave	From May to June 2021	Alpha (B.1.1.7)	34	0	0.00 (95% CI 0.00-10.15)
Fifth wave	From July to September 2021	Alpha (B.1.1.7) – Delta (B.1.617.2)	50	2	4.00 (95% CI 1.10-13.46)
Sixth wave	From October to December 2021	Delta (B.1.617.2) – Omicron (B.1.1.529) Sub-lineage BA.1	17	1	5.88 (95% CI 1.05–26.98)
Seventh wave	From January to August 2022	Omicron (B.1.1.529) Sub-lineage BA.1 – Sub-lineage BA.2 – Sub-lineage BA.5	51	9	17.65 (95% CI 9.57–30.25)
After the seventh wave	From September 2022 to May 2023	Omicron (B.1.1.529) Sub-lineage BA.5 – Sub-lineage BQ.1 – Sub-lineage BQ.1.1	57	1	1.75 (95% CI 0.31–9.29)
Total of samples			432	18	4.17 (95% CI 2.65–6.49)

## Discussion

To the author´s knowledge, this is the first study performed in Europe including a high number of ferrets from different regions of a country during the different waves of COVID-19 outbreaks. The results of this study reveal that no variation in SARS-CoV-2 seroprevalence in ferrets in Spain was detected during the different waves of COVID-19 outbreaks with the exception of the seventh wave, being BA.4 and BA.5 Omicron, the dominant virus variants. These Omicron subvariants were more transmissible but did not increase disease severity in infected humans (ECDC 2022). The presence of these subvariants could be more easily transmitted from human-to-ferret and the number of seropositive animals was higher in comparison to the remaining wave of COVID-19 outbreaks.

Different epidemiological studies in cats and dogs have been performed. These studies include sample collection during a short time or by contrast, long periods with different waves of COVID-19 outbreaks (Jairak et al. 2022; Udom et al. 2022; Villanueva-Saz et al. 2023a). However, a limited number of studies have been published in ferrets with a seroprevalence rate ranging from 0 to 1.57% (Giner et al. 2021; Kaczorek-Łukowska et al. 2023). The presence of viral RNA was also detected by molecular techniques in 6 out of the 71 of ferrets used as work animals for rabbit control in Ciudad Real (Spain) (Gortázar et al. 2021).

A previous seroepidemiological study performed in the Province of Valencia (Spain) from January to November 2020 detected 1.57% (2/127) seropositivity ferrets for SARS-CoV-2 performed with the same serological ELISA technique used in this study (Giner et al. 2021). In this study, a seroprevalence of 2.44% (95% CI 0.83–6.93) was detected from 223 ferret serum samples since December 2019 to December 2020. In this sense, a low seroprevalence level was detected in both mentioned studies including the previous seroepidemiological study realized in the Province of Valencia and the new results obtained in this study from different areas of Spain.

No question to the owners was included concerning the situation of the ferrets about living or in direct contact with confirmed COVID-19 positive humans in order to protect their private personal rights. Moreover, the presence of asymptomatic infected people during the different waves of COVID-19 outbreaks is an additional argument to avoid to include this information once the blood collection was obtained. In relation with some clinical signs detected in the seropositive ferrets, it is unlikely that are related to SARS-CoV-2 infection due to the nature of the clinical signs recorded and the absence of respiratory clinical signs (cough, rhinorrhea) in the seropositive ferrets (Kim et al. 2022). Moreover, infected ferrets by SARS-COV-2 virus under experimental conditions could not show clinical signs although virus replication was detected (Schlottau et al. 2020).

No agreement was obtained between RBD-ELISA and VNT. Seropositive ferrets detected by ELISA obtained a low value of OD units. Similar results have been described in other studies including cats (Villanueva-Saz et al. 2023a) and ferrets (Wernike et al. 2021), especially in animals with low anti-RBD IgG antibodies and a negative result obtained by VNT. In general, neutralizing activity of antibodies is evaluated with the VNT as indicator of immunity to SARS-CoV-2 infection. This test is able to evaluate the overall serum neutralizing activity including isotypes and epitope-specific antibodies to prevent virus infection in vitro conditions. In human medicine, the combination of anti-RBD IgA antibodies and anti-RBD IgG antibodies are responsible for the strongest association with antibodies neutralizing activity against SARS-CoV-2 infection. In this sense, levels of anti-RBD IgA antibodies suffer a rapid decline after SARS-CoV-2 infection suggesting that IgA isotype dominates the early neutralizing activity and this is one of the most important components of the protective humoral response (Marot et al. 2021). This finding could justify the results obtained in this study with the presence of ferrets with a positive result by ELISA but a negative result by VNT. In human medicine, there is evidence that neutralizing antibodies could decline more rapidly than anti-RBD IgG in asymptomatic infected people. In this sense, anti-RBD antibodies were still detectable, whilst, neutralizing antibodies became undetectable in some patients during a long period of time after illness onset or laboratory confirmation (Yang et al. 2022). Finally, another possible explanation of the differences detected between ELISA and VNT results could be attributed to the specificity of neutralizing antibodies obtained from ferrets during subsequent waves with the presence of other dominant variants of SARS-CoV-2 and the viral strain used to perform VNT. In our study, the Alpha variant (B.1.1.7) was used in VNT assay. This virus strain was able to detect also neutralizing antibodies against ancestral SARS-CoV-2 strain and Omicron variant (B.1.1.529) in experiments performed with sera from cats infected with the Alpha variant, in which stimulate the humoral response with an important increase in the production of neutralizing antibodies (Villanueva-Saz et al. 2023b). With the exception of 3 seropositive ferrets detected during the fifth and sixth waves when the Delta variant (B.1.617.2) was present in Spain, VNT based on the Alpha variant may not have been able to detect neutralizing antibodies due to the Delta variant. However, in cats, Delta-dominant samples showed similar titers against the Alpha variant (Tyson et al. 2023). Cross-reactivity of sera from cats infected by different variants of SARS-CoV-2 virus in VNT studies showed 3 different patterns of neutralizing antibodies using different virus variant strains. Similar cross-reactivity among variants with more or less same titers detection, slightly low cross-reactivity among variants and finally, marked low cross-reactivity with the detection of low antibody titers or even the lack of detection, similar to the patterns of neutralization observed with human serum samples (Manali et al. 2022). However, no information is available related to the pattern of neutralization based on different SARS-CoV-2 virus variants in ferrets.

The dynamics of SARS-CoV-2 antibodies in nature-infected pets including dogs, cats and ferrets have been less studied compared to studies in human medicine. The persistence of SARS-CoV-2 neutralizing antibodies has been detected up 10 months since the first positive results in dogs and cats (Decaro et al. 2022). Moreover, the kinetics of neutralizing antibodies in two infected cats showed a persistence of the neutralizing antibodies more than 16–18 months after the first positive result (Villanueva-Saz et al. 2023). In the case of ferrets, SARS-CoV-2 antibodies were detectable in a seropositive animal beyond 129 days after the first-time antibodies detection (Giner et al. 2021).

The main limitations of this study are the retrospective nature, the absence of different variants strains in the VNT and the lack of seropositive-ferrets follow-up, including the dynamics analysis of antibodies against SARS-COV-2. By contrast, the important number of samples obtained from different regions of Spain as well as the long period of study including the different waves COVID-19 outbreaks in the humans are the most important strengths of this research.

To the best our knowledge, this is the first study performed in Europe to investigate the seroprevalence of anti-SARS-CoV-2 antibodies in ferrets during the different waves of COVID-19 outbreaks in a European country. Based on the results obtained in this study, the impact of the SARS-COV-2 infection in pet ferrets in Spain was very limited and the most probably route of transmission was based on human-to-ferret transmission. Although experimental infection has demonstrated that ferrets are susceptible to SARS-COV-2 infection, this species may have a lower susceptibility in natural conditions.

### Supplementary information

Below is the link to the electronic supplementary material.

ESM 1 (DOCX 20.9 KB)

## Data Availability

The data that support the findings of this study are available from the corresponding author upon reasonable request.
