# Supplementary material for: Seroprevalence of anti-SARS-CoV-2 antibodies in household domestic ferrets (Mustela putorius furo) in Spain, 2019–2023
Source: Vet Res Commun. 2023 Aug 7;48(1):533–40. doi: 10.1007/s11259-023-10190-2 (PMC10811151; doi:10.1007/s11259-023-10190-2)
Supplement: Supplementary file 1 — (DOCX 20.9 KB) [file 11259_2023_10190_MOESM1_ESM.docx]

**Supplementary table 1.** Characteristics of the ferret population examined.

| Factor | *P* value | | | | | | |
| --- | --- | --- | --- | --- | --- | --- | --- |
|  | Age | Sex | Neutering | Cohabitation with other animals | Lifestyle | Health Status during blood collection | Wave of COVID-19 outbreak |
| SARS-CoV-2 positivity by ELISA | 0.551 | 0.425 | 0.074 | 0.235 | 0.196 | 0.999 | 0.002 |

Associations with a P value of < 0.05 were to be considered statistically significant.

**Supplementary table 2.** Characteristics of the seropositive ferrets detected in the study.

| Ferret | Optical Density detected by SARS-CoV-2 ELISA | SARS-CoV-2 micro-neutralization assay (ID50) | Classification by SARS-CoV-2 micro-neutralization | Wave of COVID-19 outbreak | Location | Age | Sex | Neutering | Cohabitation with other animals | Health status during blood collection (Cause of consultation) |
| --- | --- | --- | --- | --- | --- | --- | --- | --- | --- | --- |
| 1 | 0.290 | <1/20 | Negative | First | Madrid | N.A. | N.A. | N.A. | N.A. | N.A. |
| 2 | 0.301 | <1/20 | Negative | First | Madrid | N.A. | N.A. | N.A. | N.A. | N.A. |
| 3 | 0.280 | <1/20 | Negative | Second | Zaragoza | Adult | Female | Hormone implant | No | Sick (Weigh Loss) |
| 4 | 0.261 | <1/20 | Negative | Third | Zaragoza | Adult | Female | Hormone implant | No | Sick (Inflammatory bowel disease |
| 5 | 0.288 | <1/20 | Negative | Third | Sevilla | N.A. | N.A. | N.A. | N.A. | N.A. |
| 6 | 0.270 | <1/20 | Negative | Fifth | Valencia | Senior | Male | Intact | Yes | Sick (Kidney Disease) |
| 7 | 0.272 | <1/20 | Negative | Fifth | Sevilla | Young | Male | Intact | Yes | Sick (Gastrointestinal Disease) |
| 8 | 0.318 | <1/20 | Negative | Sixth | Valencia | Senior | Female | Hormone implant | No | Sick (Hydroureter) |
| 9 | 0.519 | <1/20 | Negative | Seventh | Bizkaia | N.A. | N.A. | N.A. | N.A. | N.A. |
| 10 | 0.276 | <1/20 | Negative | Seventh | Valencia | Adult | Female | Hormone implant | Yes | Sich (Leishmaniosis) |
| 11 | 0.285 | <1/20 | Negative | Seventh | Las Palmas | Adult | Female | Hormone implant | Yes | Healthy |
| 12 | 0.416 | <1/20 | Negative | Seventh | Navarra | Adult | Male | Hormone implant | Yes | Healthy |
| 13 | 0.300 | <1/20 | Negative | Seventh | Navarra | Senior | Female | Surgical spaying | Yes | Healthy |
| 14 | 0.343 | <1/20 | Negative | Seventh | Navarra | Aduilt | Male | Hormone implant | Yes | Healthy |
| 15 | 0.327 | <1/20 | Negative | Seventh | Alava | Senior | Female | Hormone implant | Yes | Sick (Urine stones) |
| 16 | 0.321 | <1/20 | Negative | Seventh | Bizkaia | Senior | Male | Hormone implant | Yes | Healthy |
| 17 | 0.363 | <1/20 | Negative | Seventh | Bizkaia | Senior | Male | Hormone implant | Yes | Healthy |
| 18 | 0.281 | <1/20 | Negative | After the seventh | Madrid | Senior | Female | Hormone implant | Yes | Sick (Cystine stones) |

N.A.: Not available

**Supplementary table 3.** Statistical association between SARS-CoV-2 seropositivity by ELISA and the different waves of COVID-19 outbreaks

| Factor | *P* value | | | | | | | | |
| --- | --- | --- | --- | --- | --- | --- | --- | --- | --- |
|  | First wave | Second wave | Third wave | Fourth wave | Fifth wave | Sixth wave | Seventh wave | After the seventh wave |  |
| First wave |  | 0.424 | 0.999 |  | 0.600 | 0.381 | 0.002 | 0.999 |  |
| Second wave | 0.424 |  | 0.492 |  | 0.999 | 0.999 | 0.441 | 0.474 |  |
| Third wave | 0.999 | 0.492 |  |  | 0.625 | 0.445 | 0.008 | 0.999 |  |
| Fourth wave |  |  |  |  |  |  |  |  |  |
| Fifth wave | 0.600 | 0.999 | 0.625 |  |  | 0.999 | 0.060 | 0.608 |  |
| Sixth wave | 0.381 | 0.999 | 0.445 |  | 0.999 |  | 0.438 | 0.414 |  |
| Seventh wave | 0.002 | 0.441 | 0.008 |  | 0.060 | 0.438 |  | 0.017 |  |
| After the seventh wave | 0.999 | 0.474 | 0.999 |  | 0.608 | 0.414 | 0.017 |  |  |

Associations with a P value of < 0.05 were to be considered statistically significant.
